# Clinical data on rare Sulfamethoxazole crystalluria assessed by Fourier transform infrared spectrophotometry

**DOI:** 10.1016/j.dib.2018.11.006

**Published:** 2018-11-06

**Authors:** Vincent Castiglione, Etienne Cavalier, Romy Gadisseur

**Affiliations:** Clinical Chemistry Department, CHU de Liège, University of Liège, Belgium

**Keywords:** Sulfamethoxazole, Crystalluria, Drug: adverse effect, Acute renal failure, Infrared spectrophotometry, Urine microscopy

## Abstract

The data contained in this article are related to the article entitled “Case report: Uncommon Sulfamethoxazole Crystalluria” (Castiglione et al., 2018). Sulfamethoxazole crystalluria is very rare and crystals identification is complex (de Liso et al., 2016). We identified seven patients with uncommon urine crystals that were composed of N-Acetyl-Sulfamethoxazole. Three of the patients developed an acute renal failure simultaneously to crystalluria. Hence, this data article describes the method of crystals identification thanks to infrared spectroscopy. The relevant clinical data of patients, including medical history, drug dosage and urine parameters related to the crystalluria are presented.

**Specifications table**

**Table:** 

Subject area	*Clinical biochemistry*
More specific subject area	*Drug crystalluria*
Type of data	*Figure, table*
How data were acquired	*Crystals composition was determined by Fourier Transform Infrared Spectroscopy*
	*Medical records of patients were retrospectively reviewed*
Data format	*Analyzed*
Experimental factors	*Urine sediment were dried after centrifugation*
Experimental features	*Residue was used to realize pellets for the FTIR analysis*
Data source location	*Liège, Belgium*
Data accessibility	*Data are with this article, and available as a Microsoft Excel Worksheet in supplementary data at Mendeley data*https://data.mendeley.com/datasets/z9v9rznnyk/1/files/71777564-b6f1-4e3a-91fb-970813401ec8/Table1.EX.xlsx?dl=1
Related research article	V. Castiglione, E. Cavalier, R. Gadisseur, Case report: Uncommon Sulfamethoxazole crystalluria, Clin. Biochem. (2018). doi:10.1016/j.clinbiochem.2018.05.009[1]

**Value of the data**

• The data report the largest case series of Sulfamethoxazole crystalluria.
• The description of the new crystal׳s shapes will help to suspect Sulfamethoxazole crystalluria.
• The method describes how to identify uncommon urine crystals thanks to infrared spectroscopy.
• The data will help to identify risk factors and issues of Sulfamethoxazole crystalluria in future cases.

## 1 Data

**Fig. 1:**
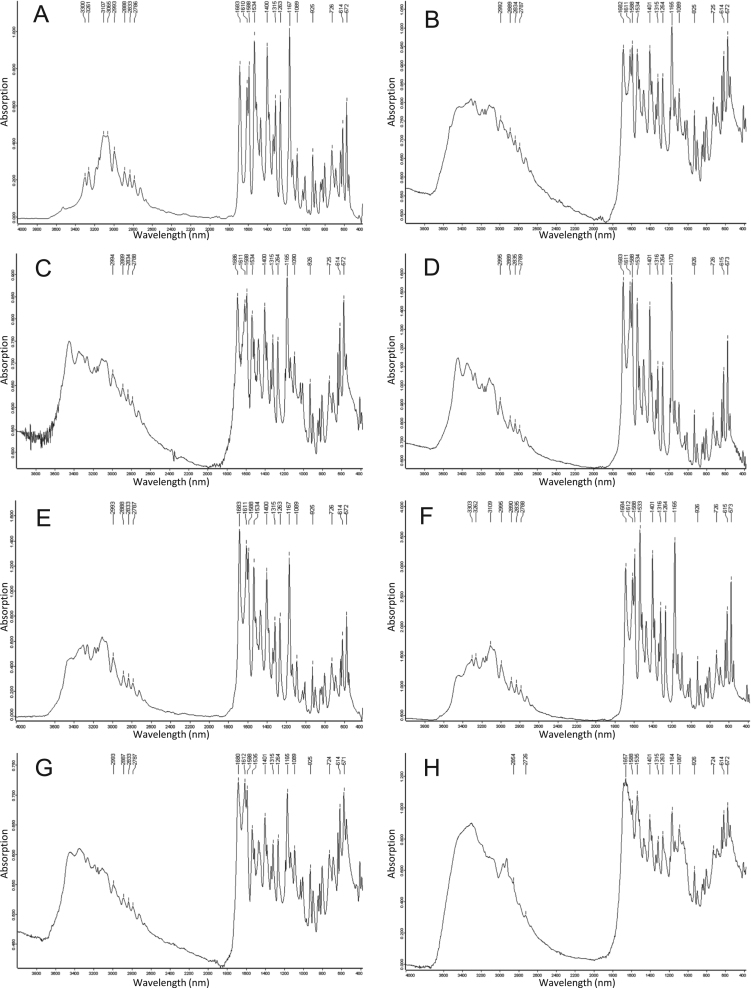
Fourier transform infrared spectra of (A) N-Acetyl-Sulfamethoxazole reference, and (B–H) dried urine residue from patient 1 to 7, respectively. The wavelengths of the main absorption peaks that are common between the samples and the reference are indicated.

Fig. 1 illustrates the infrared spectra of N-Acetyl-Sulfamethoxazole reference (NASM) and of the dried residue from patient׳s sample. The spectra of the dried residues of each patient included the absorbance peaks pattern of NASM. N-Acetyl-Sulfamethoxazole is the main metabolite of Sulfamethoxazole that can crystallize in urine [2], [3], [4], [5].

Table 1, in supplementary data (https://data.mendeley.com/datasets/z9v9rznnyk/1/files/71777564-b6f1-4e3a-91fb-970813401ec8/Table1.EX.xlsx?dl=1), describe the medical records of patients, their drug dosage, urine parameters, blood creatinine levels and their outcomes. Note that four patients had diuretic medication. Lower cotrimoxazole doses were associated to crystals with rectangular shapes, while higher doses were associated to bigger and more irregular crystals.

Images of the crystals are available in the paper “Case report: uncommon Sulfamethoxazole crystalluria” [1], where the data are discussed.

### 1.1 Supplementary data

The supplementary Table 1 is available at Mendeley data (https://data.mendeley.com/datasets/z9v9rznnyk/1/files/71777564-b6f1-4e3a-91fb-970813401ec8/Table1.EX.xlsx?dl=1).

## 2 Experimental design, materials and methods

More than 100 urine samples are analyzed daily at the University Hospital of Liège (Belgium). Between 2014 and 2017, we identified seven patients with NASM urinary crystals. The crystals were observed with the automated microscopy urine analyzer SediMAX° (Menarini, Milan, Italy). The crystals had unusual shapes, different from oxalate, uric acid, struvite or other common urine crystals. Crystals shapes included mushroom, flower, parallelepiped, truncated lozenges, thin rectangles and spheroids. In order to confirm their composition, we performed a Fourier Transform infrared spectrophotometry analysis adapted from Daudon et al. [4]. The samples were stored at room temperature and analyzed within two hours. Samples were centrifuged for 5 min at 4000 rpm. After centrifugation, the urine residue was collected on a glass and dried. The residue was then mixed with potassium bromide to make pellets that were analyzed thanks to an alpha-T infrared spectrophotometer (Bruker, Germany). The infrared spectra were then compared to spectra from a reference library (OPUS, Bruker Optics GmbH). When the pattern and wavelengths of the main peaks of the infrared spectra obtained from the samples were the same as NASM reference, it allowed identifying NASM crystalluria. The spectra also contained urea and proteins in various quantities due to other urine elements. Cotrimoxazole administration was then confirmed in all patients thanks to medical records.

We carefully reviewed medical records of each patient to check patient׳s conditions, medications, urine parameters and creatinine measurement. Urine parameters at the crystalluria onset were assessed by SediMAX and AutionMAX (Menarini, Florence, Italy).

## Appendix A Supplementary material

Supplementary Table 1 Clinical parameter of patients with N-Acetyl-Sulfamethoxazole crystalluria. COPD, chronic obstructive pulmonary disease; NA, not available; iv, intra-venous; po, per os.
