# Supplementary material for: Clinical data on rare Sulfamethoxazole crystalluria assessed by Fourier transform infrared spectrophotometry
Source: Data Brief. 2018 Nov 6;21:2033–6. doi: 10.1016/j.dib.2018.11.006 (PMC6258869; doi:10.1016/j.dib.2018.11.006)
Supplement: Supplementary file 1 — Supplementary material. [file mmc1.docx]

# Conflict of interest form

- This manuscript has not been submitted to, nor is under review at, another journal or other publishing venue.
- The authors have no affiliation with any organization with a direct or indirect financial interest in the subject matter discussed in the manuscript

Vincent Castiglione, Etienne Cavalier, Romy Gadisseur
